# Contributions of face processing, social anhedonia and mentalizing to the expression of social autistic-like traits

**DOI:** 10.3389/fnbeh.2022.1046097

**Published:** 2022-12-22

**Authors:** Johan F. Pieslinger, Joost Wiskerke, Kajsa Igelström

**Affiliations:** ^1^Division of Neurobiology, Department of Biomedical and Clinical Sciences, Linköping University, Linköping, Sweden; ^2^Center for Social and Affective Neuroscience, Department of Biomedical and Clinical Sciences, Linköping University, Linköping, Sweden

**Keywords:** autistic disorder, facial recognition, anhedonia, mentalization, phenotype, psychophysics

## Abstract

**Introduction:**

Quantitative autistic-like traits (QATs) are a constellation of traits that mirror those of clinical autism and are thought to share the same mechanisms as the condition. There is great interest in identifying the genetic and neurobiological basis of QATs, but progress is hindered by the composite nature of these clinically based constructs. Social QATs are defined according to the diagnostic criteria for autism, comprising multiple potential neural mechanisms that may contribute to varying degrees. The objective of this study was to decompose social QATs into more specific constructs, in line with the Research Domain Criteria (RDoC). We chose constructs with trait-like properties and known or suggested significance for autistic social function: (1) social anhedonia, (2) prosopagnosia (face blindness), and (3) mentalizing (attributing mental states to images of eyes). We hypothesized that these constructs may all contribute to observed variance in social QATs.

**Methods:**

We recruited 148 adults with a broad range of QATs (mean age 37.9 years, range 18–69; 50% female; 5.4% autistic) to an experimental behavioral study conducted online. We estimated social QATs using the social factor of the Comprehensive Autistic Traits Inventory. We used the Oxford Face Matching Task and the Reading the Mind in the Eyes Test to measure face matching ability and mentalizing, respectively. Social anhedonia traits were measured with the Anticipatory and Consummatory Interpersonal Pleasure Scale, and prosopagnosic traits with the 20-item Prosopagnosia Index. A combination of frequentist and Bayesian statistics was used to test the social constructs as predictors of social QATs.

**Results:**

We found that social anhedonic traits, prosopagnosic traits, and face matching performance were likely predictors of social QATs, whereas mentalizing showed limited contribution.

**Conclusion:**

The findings support prosopagnosic and anhedonic traits, but not mentalizing deficits, as dimensional predictors of individual differences in social function across the autistic spectrum. Further, the study strongly suggests that social reward systems and face processing networks play significant and independent roles in autistic-like social function.

## 1 Introduction

Quantitative autistic-like traits (QAT)—which comprise differences in social interaction and communication, and rigid/repetitive behaviors (RRB)—exist on a continuum across the entire population. While these characteristics are elevated in individuals with a known genetic load for autism, such as family members of autistic individuals (Hurley et al., 2007), they are not specific to this group (Lord and Bishop, 2021). However, the genetic mechanisms of QATs in the general population appear to overlap with those in clinical autism (Landry and Chouinard, 2016; Thomas et al., 2022).

Quantitative autistic-like trait questionnaires, such as the Autism Quotient (Baron-Cohen et al., 2001b), Broad Autism Phenotype Questionnaire (Hurley et al., 2007) or Comprehensive Autistic Trait Inventory (CATI; English et al., 2021), have been used extensively in research studies to identify biological correlates of autism-relevant characteristics. Such research is always limited by the biological validity of the studied constructs: if there is more than one mechanism, the results will inevitably have high variance. This obstacle is likely to be very significant for QATs, which represent a neurophysiologically heterogeneous collection of behaviors and preferences. Several studies have suggested that QATs can be fractionated into genetically dissociable social and non-social domains (Ronald et al., 2006; Dworzynski et al., 2009; Warrier et al., 2019). However, although these sub-domains in isolation are more circumscribed than the total QATs, they still represent extremely broad constructs.

In the meantime, our understanding of neural mechanisms underlying human behavior has advanced to a more fine-grained level. This is reflected in the NIMH’s Research Domain Criteria (RDoC) initiative, which aims to identify the most fundamental genetic, phenotypic, neurobiological, and behavioral building blocks of human function and dysfunction (Cuthbert and Insel, 2013; Morris et al., 2015; Kozak and Cuthbert, 2016). RDoC constructs, developed by expert workgroups, are intended to be associated with distinct neural circuits and not overlap with other constructs.

From an RDoC-based perspective, socio-communicative QATs are made up of a combination of social, cognitive, and affective functions (Tiede and Walton, 2021). Some of these functions might show polygenic inheritance patterns and population distributions similar to the QATs, and some might potentially vary bimodally in a way more consistent with categorical dysfunction. These functions can all be conceptualized as transdiagnostic constructs that are narrower and neurally more specific than the classical QATs. They show natural variation in the general population, but they also tend to be related to multiple psychiatric conditions. In the current study, we focus on three fundamental building blocks of social function: (1) social motivation, (2) face perception, and (3) mentalizing.

### 1.1 Social motivation and social anhedonia

Social stimuli function as intrinsic reinforcers, resulting in a strong preference for social stimuli from the very beginning of life. This attentional bias drives further social stimulation and supports the development of social brain circuits. Social interaction and affiliation are also associated with a hedonic “liking” response, which can be estimated in adults through self-report measures of social anhedonia. Social anhedonia differs from social anxiety which is an increase in negative affect associated with social situations rather than a lack of positive affect. Individual differences in social motivation and pleasure may be caused by variations in brain networks underlying reward processing and social affiliation. Social reward responsiveness, reward valuation and reward learning involve the amygdala and mesolimbic circuits, as well as the ventromedial prefrontal and orbitofrontal cortex (Bottini, 2018). The “liking” of social stimuli also involves signaling by neuropeptides such as oxytocin and endogenous opioids in distributed areas (Berridge and Robinson, 2016). In the RDoC matrix, social anhedonia is currently separated from general reward processing by its inclusion in the Social Processes domain within the construct of Affiliation and Attachment.

A systematic review of reward processing in autism found consistently decreased social reward learning in autistic children and adults (Bottini, 2018), and a meta-analysis of fMRI studies suggested differences in neural responses during processing of many types of rewards (Clements et al., 2018). Self-reported social, but not physical, pleasure was decreased in autistic male adolescents (Chevallier et al., 2012a). Social anhedonia has been suggested to be an endophenotype of autism, based on elevated levels in parents of autistic individuals (Berthoz et al., 2013) and linear correlation with QATs also after correcting for non-social anhedonia (Novacek et al., 2016).

### 1.2 Processing of face identity and prosopagnosia

A fundamental prerequisite for social affiliation and attachment is our ability to recognize conspecifics. The face processing network, consisting of the fusiform face area, inferior frontal gyrus and the amygdala, is necessary for face recognition and face memory. Face recognition is heavily dependent on visuospatial perception and attention, which could be considered part of the RDoC construct Perception, in the domain of Cognitive Systems. The development of the highly specialized face processing areas and associated networks requires practice. In autism, early differences in social motivation may decrease visual orienting to faces (Jones and Klin, 2013), likely hampering the development of face expertise (Yrttiaho et al., 2022).

Roughly a third of autistic individuals exhibit prosopagnosia (face blindness; Minio-Paluello et al., 2020). Prosopagnosia refers to the inability to accurately process or recognize faces. Autism studies have reported deficits in both low-level face perception and the ability to encode faces in memory (Dalrymple et al., 2014; Dalrymple and Palermo, 2016; Minio-Paluello et al., 2020; Stantić et al., 2022b). The face processing network has also been shown to be affected in individuals with autism and their parents (Haxby et al., 2000; Wilson et al., 2010; Joseph et al., 2015; Yucel et al., 2015; Liu et al., 2021). The genetic basis of prosopagnosia was found to overlap with that of social QATs (Lewis et al., 2018). Several studies have found correlations between QATs and performance in various face processing paradigms, although findings have been mixed (Davis et al., 2011, 2017; Hedley et al., 2011; Verhallen et al., 2017; Rigby et al., 2018).

### 1.3 Mentalizing

Another construct important for successful social interaction is the ability to discern emotional states in other individuals. This is often referred to as mentalizing, and is classically thought to contribute to the autistic phenotype (Tiede and Walton, 2021). As faces constitute salient social cues, a common test of mentalizing is the Reading the Mind in the Eyes Test (RMET). In the RMET, participants infer emotional states based on photographs of eyes (Baron-Cohen et al., 2001a). This requires perception and integration of very subtle facial cues and can resolve individual differences in non-clinical populations (Baron-Cohen et al., 2001a; Turner and Felisberti, 2017). Mentalizing in the RMET activates social brain regions, in particular the left inferior frontal gyrus, middle cingulate cortex, and posterior superior temporal sulcus (Adams et al., 2010; Schurz et al., 2014). Variations in performance were correlated with connectivity between frontoparietal control network and the default mode network (van Buuren et al., 2021). In the RDoC matrix, mentalizing is represented in the Social Processes domain under the construct Perception and Understanding of Others (subconstruct Understanding Mental States). While some studies have found atypical RMET performance in autism, the evidence for a dimensional and genetically based relationship with QATs is relatively weak (Baron-Cohen and Hammer, 1997; Baron-Cohen et al., 2001a,^2015^; Losh et al., 2009; Miu et al., 2012; Tajmirriyahi et al., 2013).

### 1.4 Aim of the study

The composite nature of QATs gives it high clinical relevance but is neurobiologically non-specific. Neurally based constructs that contribute mechanistically to the autistic phenotype should predict at least some of the variance in QAT expressions. The rationale of this study is based on the RDoC-compatible assumption that the QAT score is the sum of variations in more specific transdiagnostic functional components (Cuthbert, 2022). Identification of RDoC-based measurable predictors of the QATs will facilitate further study of the biological mechanisms of social interaction differences in autism. This study represents one step in that direction, testing behaviors mediated by the three distinct but partially overlapping brain networks described above, that span several RDoC domains and constructs. We used web-based perception tests of face matching and mentalizing, together with reliable self-report measures of social anhedonia, prosopagnosia and QATs, to test the contribution of these RDoC constructs to autistic-like features.

## 2 Materials and methods

Throughout this article, we have chosen to describe autism and autistic differences mainly using identity-first language (autistic person rather than person with autism) and non-pathologizing terminology, in line with current recommendations based on the preference of relevant stakeholders (Monk et al., 2022).

### 2.1 Recruitment and procedure

Experiments were conducted between January and March 2022. Participants (*N* = 250) were invited *via* Prolific^1^ (Peer et al., 2021). The same participants first participated in a different study (Bang and Igelström, 2022). Background questions were asked using Qualtrics CoreXM (Qualtrics Ltd., WA, USA) and were followed by two questionnaires not used in the current study (Broad Autism Phenotype Questionnaire and Glasgow Sensory Questionnaire). All other study components were run using the Gorilla Experiment Builder,^2^ which utilizes JavaScript to run experiments in the participant’s own browser (Anwyl-Irvine et al., 2020).

The test battery was adminstered online and contained measures of QATs (CATI; English et al., 2021), social anhedonia (The Anticipatory and Consummatory Interpersonal Pleasure Scale, ACIPS; Gooding and Pflum, 2014b), mentalizing (RMET; Baron-Cohen et al., 2001a), prosopagnosia (The Prosopagnosia Index, PI20; Shah et al., 2015), and lower-level face matching performance (the Oxford Face Matching Test, OFMT; Stantić et al., 2022a).

**FIGURE 1:**
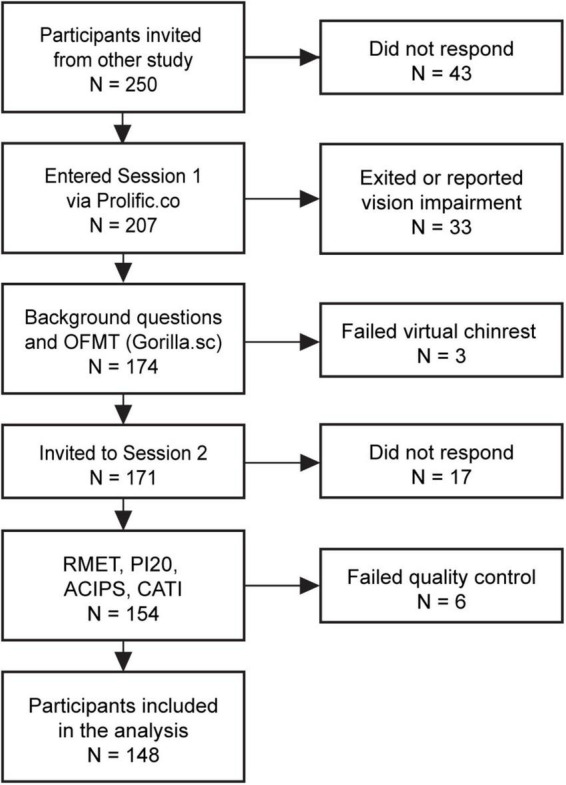
Illustration of the experiment flow and reasons for attrition or exclusion. Invitation of participants consisted of advertising a study on Prolific with a built-in visibility filter that was based on participation in a different experiment; thus, no interactions occurred between researchers and participants at this step. See section “Materials and methods” for quality control criteria for tasks and questionnaires. ACIPS, Anticipatory and Consummatory Interpersonal Pleasure Scale; OFMT, Oxford Face Matching Task; PI20, 20-item Prosopagnosia Index; RMET, Reading the Mind in the Eyes Test.

After recruitment from the other study, the experiments were conducted at two separate occasions, as illustrated in the flow diagram in Figure 1. Session 1 consisted of the OFMT and background questions on ethnicity and prosopagnosia. Session 2 started with the RMET and continued with the PI20, the ACIPS, and CATI in randomized order. A Virtual Chinrest Task (VCRT) was included before the OFMT and RMET to allow for scaling of stimuli to the desired visual angle (VA; see below).

For the original study, the recruitment was set up to aim for a 50-50 gender distribution, age range 18–70 years, English as their first language, and at least 10 previous submissions at 100% approval rate on Prolific. For Sessions 1 and 2, a preselection filter was set to include participants who had participated in the previous part. Participants with schizophrenia, psychotic disorder, neurological disorders, or vision impairment were excluded. There was gradual attrition between the different stages (Figure 1) with *N* = 175 completing the face matching task, and *N* = 148 completing all experiments.

According to Swedish regulations (law: 2003:460), a study must undergo ethical review if it collects personal data. Studies that do not fulfill this requirement do not undergo ethical review; thus, this study was not reviewed by an ethical review board. All procedures were in strict concordance with the Declaration of Helsinki and the design was based on similar studies that passed through institutional ethical review in the United States (KI). Participants provided digital informed consent at the beginning of each part and were aware of the progression between the experiments. The participants were reimbursed through Prolific for their time.

### 2.2 Measures

#### 2.2.1 Background questions

The background section contained questions about age, gender, and country of residence. Participants were also asked about primary sensory deficits and psychiatric and neurodevelopmental conditions. In Session 1, we included a question about ethnicity (White/Caucasian versus Other) for use as a covariate to control for own-race bias in OFMT and RMET analyses (Meissner and Brigham, 2001; McKone et al., 2017; Wong et al., 2020), because these tasks used only Caucasian faces (Baron-Cohen et al., 2001a; Stantić et al., 2022a). Participants also received the Yes/No question “Do you think you have any problem with recognizing faces?”

#### 2.2.2 The Comprehensive Autistic Trait Inventory

The CATI quantifies QATs within the classical domains of social, communicative, and rigid traits, as well as sensorimotor differences (English et al., 2021). The questionnaire consists of 42 statements regarding an individual’s personal experience, scored on a Likert scale from 1 (Definitively Agree) to 5 (Definitively Agree). There are six subscales that contribute independently to predicting autistic status (Social Interactions, Communication, Social Camouflage, Cognitive Rigidity, Repetitive Behaviors and Sensory Sensitivity). The three social subscales can also be combined into a Social Factor, and the remaining subscales form the Restricted/Repetitive Behaviors (RRB) Factor. Total scores range from 42 to 210. This study used CATI-Social Factor to quantify total sociocommunicative QATs. The internal reliability of CATI-Social Factor in the current study was high (Cronbach’s α = 0.942 [0.927–0.954]).

#### 2.2.3 The 20-item Prosopagnosia Index

The PI20 provides a reliable measure of face recognition (Shah et al., 2015; Tsantani et al., 2021). It consists of 20 statements scored on a 5-point Likert scale ranging from Strongly Agree to Strongly Disagree (Shah et al., 2015). The total score ranges from 20 to 100 points, with higher scores indicating greater prosopagnosic traits. The PI20 score is correlated with performance across several paradigms investigating face perception and face memory (e.g., Cambridge Face Memory Test, Glasgow Face Matching Test), as well as with autistic trait scores (Shah et al., 2015; Gehdu et al., 2022; Stantić et al., 2022a). It was also correlated with the OFMT in most, but not all, reports (Stantić et al., 2021, 2022a,2022b). The internal reliability of the PI20 in the current study was high (Cronbach’s α = 0.942 [0.928–0.953]).

#### 2.2.4 Virtual Chin-Rest Task

We used the VCRT to scale stimuli based on participants’ distance to the screen (Li et al., 2020). The task calculated the logical pixel density of the participant’s screen (derived by asking the participant to resize a box to fit a standard credit card) and estimated the viewing distance with a blind spot task. The blind spot task consisted of five trials, in which the participant covered their right eye and focused with the left eye on a fixation point. In each trial, a red circle moved leftward on the screen and the participant hit the SPACE button when the ball appeared to vanish. The Gorilla experiment was programmed to force participants to repeat the task if an embedded variable signaled excessive variability between the five trials. The embedded variable represented the largest difference between the averaged calculated viewing distance and single-trial results. The threshold was set at a discrepancy of 5 cm. After three failed VCRTs, the participant was excluded. Three participants were excluded based on this criterion.

#### 2.2.5 The Anticipatory and Consummatory Interpersonal Pleasure Scale

The ACIPS is a 17-item self-report questionnaire that reliably measures social anhedonia in nonclinical samples (Gooding and Pflum, 2014a,b). The questionnaire measures the extent of pleasure a participant feels in interpersonal scenarios. The questions are posed as statements about the participant and the participant answers based on how true the statement is for them. The answers are structured using a 6-point Likert scale and total scores range from 17 to 102, with higher scores indicating higher levels of social pleasure (lower anhedonia). The total scale score was used as a dimensional measure of social anhedonia. The internal reliability of the total ACIPS was high (Cronbach’s α = 0.922 [0.903–0.938]).

#### 2.2.6 The Reading the Mind in the Eyes Test

The RMET revised version (Baron-Cohen et al., 2001a) comprised 36 trials and was presented in the participant’s browser. On each trial, the participants were asked to make a forced choice between four mental states (e.g., “jealous,” “playful”) to indicate the emotional expression of a photograph of the eye region of an actor. Accuracy was calculated as the number of correct responses divided by the total number of trials, converted to a percentage.

#### 2.2.7 The Oxford Face Matching Task

The OFMT measures lower-level face matching and has been validated against prosopagnosic and super-recognizer populations, and imposes minimal memory demands on participants (Stantić et al., 2022a). The stimuli were shared by the developers of the OFMT through the Gorilla platform. All face stimuli were in grayscale and portrayed Caucasian males and females ranging from 18 to 70 years of age. The faces were photographed from the front.

Each trial started with a fixation crosshair for 250 ms, followed by two face stimuli presented simultaneously for 1600 ms to the left and right of the fixation cross. Face stimuli were scaled to a width of 20° VA, and the center of the stimuli were positioned at 20° VA from the fixation cross. Participants were asked to make a forced choice by button press, to indicate whether the two faces belong to the same person or to different people. Upon responding, participants proceeded automatically to the next trial. There were 200 trials, including 50% same-face and 50% different-face trials counterbalanced across 4 blocks. Within each trial type, there were 20 difficulty bins, originally derived from average similarity ratings from three different face recognition AIs. For analysis, we binned these further into five bins with 20 trials per bin per trial type (difficulty D1–D5).

There were 12 randomly interspersed attention checks comprising same-face trials (identical images) and different-face trials (different genders). Participants were excluded if they failed > 2 attention checks (*N* = 0), or if they had an accuracy < 70% at the easiest difficulty level on either same-face trials or different-face trials (*N* = 6).

**FIGURE 2:**
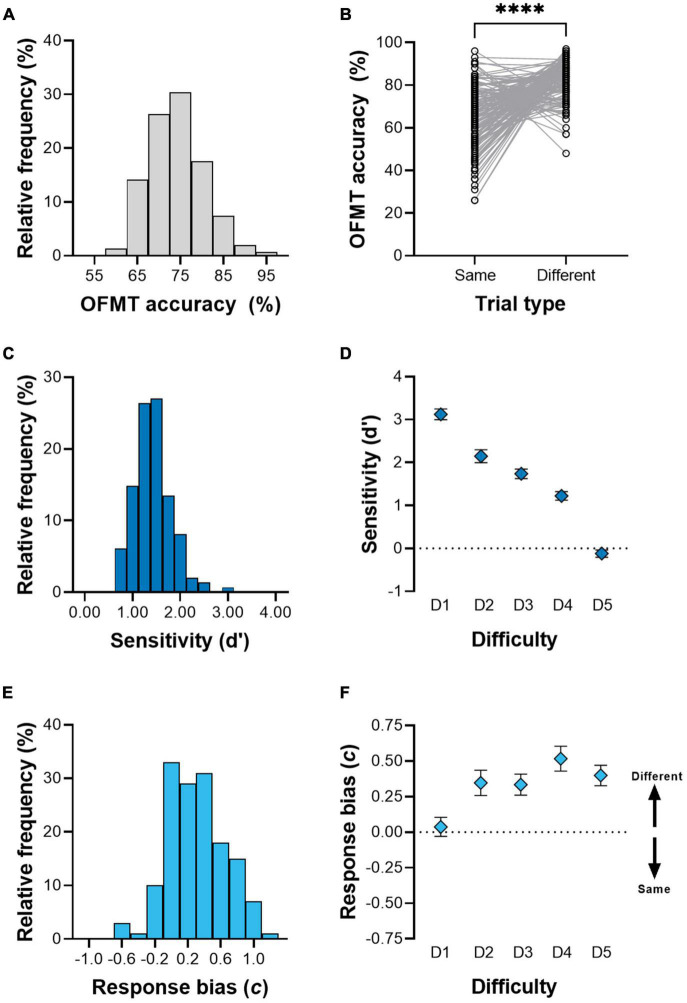
Accuracy, sensitivity, and response bias in the OFMT. **(A)** Distribution of overall accuracy in the OFMT across trial types and difficulties. **(B)** Accuracy difference between trial types. **(C)** Distribution of d′ values. **(D)** Average sensitivity (d′) (95% C.I.) as a function of difficulty. **(E)** Distribution of response biases. **(F)** Average (95% C.I.) response bias as a function of difficulty. ^****^P = 2.6 × 10^–19^.

We found a large difference in accuracy between same-face and difference-face trials (Figure 2), which made individual differences in the overall OFMT accuracy difficult to interpret. Therefore, we used signal detection theory to quantify performance and response bias, which better models the decision-making processes underlying OFMT performance (Stanislaw and Todorov, 1999). Signal detectability (leading to correct identification of matching faces) was indexed as the variable d′, which is the distance between signal and noise means in standard deviation units, i.e., the difference between the z-scored hit rate (Φ(H)) and false alarm rate (Φ(F)) (Equation 1). To allow for calculation of these values, we first replaced probabilities of 0 with 0.01, and probabilities of 1 with 0.99.


(1) $${\text{d}}^{\prime }=\Phi \,\left(\text{H}\right)-\Phi \,\left(F\right)$$

The response bias *c* was calculated according to Equation 2, with positive values signifying a greater bias toward responding “Different person,” and vice versa.


(2) c=(Φ⁢(H)+Φ⁢(F))/2

The total d′ and c across all 200 trials were used as dependent variables in analyses concerning QAT. We also calculated d′ and *c* for the five difficulty bins for Figure 2.

### 2.3 Statistics

Data were preprocessed with in-house MATLAB scripts, and statistical analyses were done in JASP v.0.16.2 (JASP-Team, 2022). Shapiro–Wilks tests were used to test for normality and Mann–Whitney tests to test for gender differences. The difference in OFMT accuracy between trial types was tested with a paired *t*-test and represented as mean ± SD. Non-parametric Friedman tests with pairwise Conover *post-hoc* tests were used to evaluate how d′ and c varied with difficulty level (D1–D5).

Frequentist linear regression models were used for null hypothesis testing of prediction of social QATs by single social constructs while correcting for age, gender (male = 1), ethnicity (white/Caucasian = 1), depression, anxiety, and attention deficit/hyperactivity disorder. Assumption checks included correlation analyses to check for nonlinear relationships, variance inflation factors to check for collinearities, and inspection of Q-Q plots, residual vs. predicted relationships, and residual distributions. There were no multivariate outliers (defined as Cook’s distance > 1).

Logistic regression was used to test for associations between social constructs and diagnosed autism. To facilitate comparisons of odds ratios, the social predictors were z-scored. As the outcome was infrequent (5.4%), we did not include covariates. The results should be viewed as preliminary given the small number of autistic participants. We created five logistic regression models, each one with autism as the dependent variable and a z-scored social construct as the independent variable. Odds ratios with 95% CIs were calculated for all social constructs.

We used Bayes factor hypothesis tests in JASP, to quantify evidence for and against the value of adding predictors to models of social QATs. Assumption checks included correlation analyses to check for nonlinear relationships, variance inflation factors to check for collinearities, and inspection of Q-Q plots and residual vs. fitted relationships. Prior distributions for regression parameters were determined using the Jeffreys-Zellner-Siow prior with an r scale of 0.354 (JASP’s default). Prior model probabilities were set to be uniform. Bayesian Adaptive Sampling was used to sample without replacement from the space of models. Robustness of the results to different priors was explored by running the analysis with other prior distributions and inspecting the results. Bayes Factors (BF) were interpreted according to Lee and Wagenmakers’ classification scheme, which suggest BF ranges for anecdotal (BF = 0.33–3), moderate (BF of 0.1–0.33 or 3–10), strong (BF of 0.03–0.1 or 10–30), very strong (BF of 0.01–0.03 or 30–100), and extreme (BF of <0.01 or >100) evidence for a hypothesis (Quintana and Williams, 2018). Model-averaged parameter estimates and credible intervals are reported.

## 3 Results

Table 1 shows demographic details of the 148 included participants. The sample was gender-balanced and aged 37.9 years on average (range 18–69). The majority of the participants resided in the United Kingdom or United States, and 89% of the sample reported White/Caucasian ethnicity. Seventeen participants (11.5%) responded “yes” to the question “Do you think you have any problem with recognizing faces?” and 13 (8.8%) scored above the prosopagnosia threshold on the PI20. Out of the participants who did not endorse any subjective problems, 93% scored below the prosopagnosia threshold on the PI20.

**TABLE 1:** Participant characteristics.

	*N*	%
**Gender**		
Male	72	48.6
Female	74	50.0
Non-binary/Other	2	1.4
**Ethnicity**		
White/Caucasian	131	88.5
Other	16	10.8
Not provided	1	0.7
**Country of residence**		
United Kingdom	90	60.8
United States	34	23.0
Canada	14	9.5
Ireland	4	2.7
Australia	3	2.0
New Zealand	3	2.0
**Conditions**		
Autism spectrum disorder	8	5.4
Attention deficit/Hyperactivity disorder	9	6.1
Mood disorder	69	46.6
Anxiety disorder	72	48.6

### 3.1 Face matching performance

We first validated that the OFMT results were in line with those described in previous studies (e.g., normal distribution and effects of difficulty level on accuracy; Stantić et al., 2022a). The overall OFMT accuracy was approximately normally distributed and captured a large range of performance (Figure 2A). However, we observed a large significant difference in accuracy between same-face trials and different-face trials (64.8 ± 0.147 % versus 82.9 ± 0.093 %; t_147_ = –10.4, *p* = 2.6 × 10^–19^; Figure 2B), and there was a negative relationship between the accuracy on same-face trials and accuracy on different-face trials (*r* = –0.531, *p* = 3.7 × 10^–12^). To test whether this reflected an anomaly in our data, we downloaded the large dataset used by Stantić et al. (2021), which was available online (accessed on July 5, 2022).^3^ In this previous dataset, we found the same negative correlation between same- and different-face trials (*r* = –0.487, *p* = 9.2 × 10^–36^). These findings indicate that there may be a strong response bias toward perceiving identities as different, possibly arguing against pooling accuracy across same- and different-face trials.

To understand this response pattern, we used signal detection theory (SDT) to better distinguish between the sensitivity to matching faces and the degree of response bias (Stanislaw and Todorov, 1999). We calculated the sensitivity, d prime (d′), for identifying face matches, and the bias *c* to quantify the tendency to label faces as mismatches. This method fully characterizes task performance by considering the number of hits (proportion correct responses in same-face trials) and false alarms (proportion incorrect responses in different-face trials) We found a slightly skewed distribution of d′ (Figure 2C) with most values falling between 1 and 3. d′ progressively dropped as the difficulty increased (Figure 2D). A non-parametric Friedman test showed a significant main effect of Difficulty (χ^2^(4) = 482.7, *p* = 3.8 × 10^–103^), and Conover *post-hoc* tests showed differences between all pairs. Most participants showed a response bias favoring the conclusion that faces were different (Figure 2E). This bias was absent on average at the lowest difficulty (D1 in Figure 2F) but increased at the higher difficulties (D2–D5 in Figure 2F). There was a significant effect of Difficulty also for the response bias (χ^2^(4) = 130.4, *p* = 3.2 × 10^–27^), and *post-hoc* tests showed that D1 was different from all other difficulties, and D4 was significantly different from D2, D3, and D5. Taken together, these results indicate that the sensitivity measure d′ reflects behavior in a less biased way than the overall OFMT accuracy. Thus, we used d′ and *c* as measures of face matching performance in all analyses in the current study.

### 3.2 Associations of social constructs with social QATs

**FIGURE 3:**
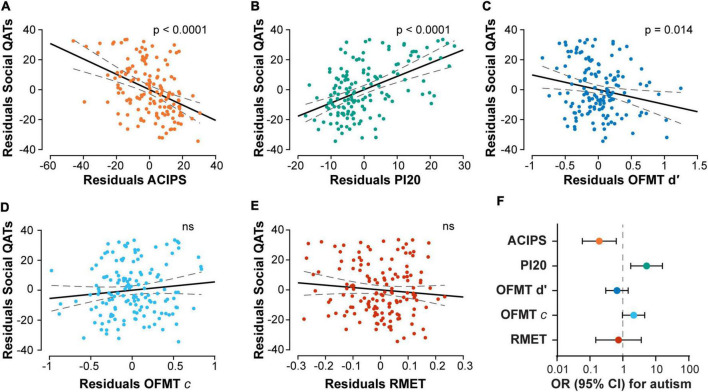
Associations of social constructs with social QATs and diagnosed autism. Scatter plots show the partial correlations for social constructs, correcting for age, gender, ethnicity, and comorbidities. *P*-values are those of the individual coefficients; ns indicates that the model was not significant (full models are reported in Supplementary Tables 1–3 and in the section “Results”). **(A)** Social anhedonia quantified with the ACIPS; lower scores indicating higher anhedonia. **(B)** Prosopagnosic traits quantified with the PI20; higher scores correspond to higher prosopagnosia. **(C)** Face matching sensitivity quantified with the OFMT d′. **(D)** Face matching response bias quantified with the bias c in the OFMT. **(E)** Mentalizing measured with the RMET. **(F)** Odds ratios and 95% confidence intervals for prediction of autism diagnosis by the social constructs. ACIPS, Anticipatory and Consummatory Interpersonal Pleasure Scale; OFMT, Oxford Face Matching Task; PI20, 20-item Prosopagnosia Index; RMET, Reading the Mind in the Eyes Test.

Social anhedonia, prosopagnosia, mentalizing, and face matching sensitivity and bias were first tested one at the time as predictors of social QATs while correcting for age, gender, ethnicity, mood disorders, anxiety disorders, and ADHD (Figure 3 and Supplementary Tables 1–3). Simple logistic regressions were also performed to test the relationship with diagnosed autism for comparison (Figure 3F; see section “2. Materials and methods”).

The distribution of social anhedonia traits (total ACIPS) was slightly skewed toward higher scores (lower social anhedonia; *W* = 0.968, *p* = 0.002). There were no gender differences (*U* = 3037.0, *p* = 0.249). Social QATs were strongly predicted by higher social anhedonia after controlling for confounding variables (Figure 3A; standardized β = –0.418; see Supplementary Table 1 for full regression results). Social anhedonia also strongly predicted an autism diagnosis (Nagelkerke *R*^2^ = 0.151, χ^2^(146) = 7.853, *p* = 0.005; Figure 3F).

Prosopagnosic traits (PI20) were positively skewed (*W* = 0.913, *p* = 9.0 × 10^–8^) and showed no gender differences (*U* = 3014.5, *p* = 0.286). Participants who had answered “yes” to the question “Do you think you have any problem with recognizing faces?” scored 63 ± 8 (median ± MAD), whereas those who said “no” scored 44 ± 4 (*U* = 398.0, *p* = 1.7 × 10^–5^). Prosopagnosia strongly predicted social QATs after controlling for confounding variables (Figure 3B; standardized β = –0.298; Supplementary Table 2). Prosopagnosia strongly predicted an autism diagnosis (Nagelkerke *R*^2^ = 0.173, χ^2^(146) = 9.052, *p* = 0.003; Figure 3F).

The distribution of OFMT d′ looked nearly normal except for a thin tail corresponding to a smaller number of high scorers (*W* = 0.972, *p* = 0.004). Male participants showed significantly worse sensitivity to matching faces than females (*U* = 3485.0, *p* = 0.004). The bias *c* was normally distributed (*W* = 0.990, *p* = 0.399) and showed no gender differences. The OFMT d′ predicted social QATs when correcting for other variables (Figure 3C and Supplementary Table 3), but it did not significantly predict an autism diagnosis (Nagelkerke *R*^2^ = 0.022, χ^2^(146) = 1.130, *p* = 0.288). Using the response bias in an equivalent model did not find a significant regression equation (*R*^2^ = 0.095, *F*_7,139_ = 2.080, *p* = 0.050; Figure 3D; the borderline significance was due to the influence of age on social QAT). The bias did not significantly predict an autism diagnosis, but there was a trend toward increased bias (Nagelkerke *R*^2^ = 0.074, χ^2^(146) = 3.8147, *p* = 0.051; Figure 3F).

Accuracy in the RMET ranged from 50 to 94%, with a slightly negatively skewed distribution (*W* = 0.959, *p* = 1.2 × 10^–4^) and no significant gender differences (*U* = 3030, *p* = 0.258). RMET did not predict social QATs after correcting for confounders (*R*^2^ = 0.092, *F*_7,139_ = 2.007, *p* = 0.058; Figure 3E), and it did not predict an autism diagnosis (Nagelkerke *R*^2^ = 0.003, χ^2^(146) = 0.134, *p* = 0.715; Figure 3F).

### 3.3 Bayesian model comparison

The results above suggested that prosopagnosia, face matching and social anhedonia were all predictive of social QATs. However, the analyses did not show whether these predictors were independent of each other. To test whether social anhedonia, face matching, and prosopagnosia had an additive effect on social QATs, we used a Bayesian regression approach to evaluate the strength of evidence for and against the value of adding predictors to the model. We separated face matching from self-reported prosopagnosia in this analysis because the constructs partially overlapped. Face matching performance isolates the lower-level perceptive face processing from higher-level processing such as face memory, whereas the prosopagnosia questionnaire measures both. This partial overlap was evident in our data, as prosopagnosia and face matching performance were correlated with each other (Spearman’s rho = –0.300, *p* = 2.1 × 10^–4^). While face matching showed a relatively small contribution in the regression analyses above (Supplementary Tables 2, 3), the OFMT has the important advantage of being objective as well as neurally specific. Therefore, we first tested models with OFMT measures, excluding prosopagnosia (PI20), and then switched to PI20 in the second analysis. In both analyses, we used a uniform prior distribution and the null model contained age, gender, ethnicity, mood disorder, anxiety disorder, and ADHD.

In the first analysis (Table 2), social anhedonia and the OFMT variables sensitivity (d′) and bias (c) provided eight (2^3^) possible models to test against our data. We found that the best model contained social anhedonia together with face matching sensitivity (*R*^2^ = 0.285), with extremely strong evidence in favor of this model (BF = 524007.202). There was anecdotal evidence against adding the OFMT bias c as a predictor (BF_10_ = 0.335 compared with best model). Social anhedonia alone as a predictor showed worse predictive adequacy than the model containing social anhedonia and face matching sensitivity (BF_10_ = 0.147).

**TABLE 2:** Model comparison for predictors of social QATs (face matching performance and social anhedonia).

Models	P(M)	P(M|data)	BF_M_	BF_10_ compared with best model	BF_10_ compared with null model	R^2^
Social anhedonia + face matching sensitivity (d′)	0.125	0.651	13.071	1.000	524007.202	0.285
Social anhedonia + face matching sensitivity (d′) + face matching bias (c)	0.125	0.218	1.955	0.335	175657.943	0.285
Social anhedonia	0.125	0.096	0.743	0.147	77233.029	0.249
Social anhedonia + face matching bias (c)	0.125	0.034	0.250	0.053	27718.083	0.250
Face matching sensitivity (d′)	0.125	6.304 × 10^–6^	4.413 × 10^–5^	9.681 × 10^–6^	5.073	0.121
Face matching sensitivity (d′) + face matching bias (c)	0.125	3.397 × 10^–6^	2.378 × 10^–5^	5.216 × 10^–6^	2.733	0.125
Null model	0.125	1.243 × 10^–6^	8.700 × 10^–6^	1.908 × 10^–6^	1.000	0.082
Face matching bias (c)	0.125	1.049 × 10^–6^	7.344 × 10^–6^	1.611 × 10^–6^	0.844	0.094

P(M) indicates the prior probability of the model. P(M|Data) is the posterior probability of the model compared to all other models where the sum of the variable across all models equals 1. BF_M_ compares the probability of a model to the average probability of all other models. BF_10_ compares the probability of the model to the null or best model. R^2^ indicates the proportion of variance explained by the model. All models include age, gender, ethnicity, mood disorder, anxiety disorder, and attention deficit/hyperactivity disorder. All predictors were z-scored. Social anhedonia was quantified with the Anticipatory and Consummatory Interpersonal Pleasure Scale, and face matching sensitivity d′ and bias c were measured through the Oxford Face Matching Task.

In the second analysis (Table 3), in which prosopagnosia (PI20 score) was included instead of face matching performance, there were four (2^2^) possible models to compare. The best model contained social anhedonia together with prosopagnosia (*R*^2^ = 0.439). The model containing both predictors was much more likely given the data than the models containing one of them (BF_10_ < 0.0001; Table 3).

**TABLE 3:** Model comparison for predictors of social QATs (self-reported prosopagnosia and social anhedonia).

Models	P(M)	P(M|data)	BF_M_	BF_10_ compared with best model	BF_10_ compared with null model	R^2^
Social anhedonia + prosopagnosia	0.250	1.000	216315.587	1.000	2.714 × 10^+12^	0.439
Prosopagnosia	0.250	1.384 × 10^–5^	4.152 × 10^–5^	1.384 × 10^–5^	3.756 × 10^+7^	0.319
Social anhedonia	0.250	2.846 × 10^–8^	8.537 × 10^–8^	2.846 × 10^–8^	77233.029	0.249
Null model	0.250	3.685 × 10^–13^	1.105 × 10^–12^	3.685 × 10^–13^	1.000	0.082

P(M) indicates the prior probability of the model. P(M|Data) is the posterior probability of the model compared to all other models where the sum of the variable across all models equals 1. BF_M_ compares the probability of a model to the average probability of all other models. BF_10_ compares the probability of the model to the null or best model. All models include age, gender, ethnicity, mood disorder, anxiety disorder, and attention deficit/hyperactivity disorder. All predictors were z-scored. Social anhedonia was quantified with the Anticipatory and Consummatory Interpersonal Pleasure Scale, and prosopagnosia was quantified with the 20-item Prosopagnosia Index.

**FIGURE 4:**
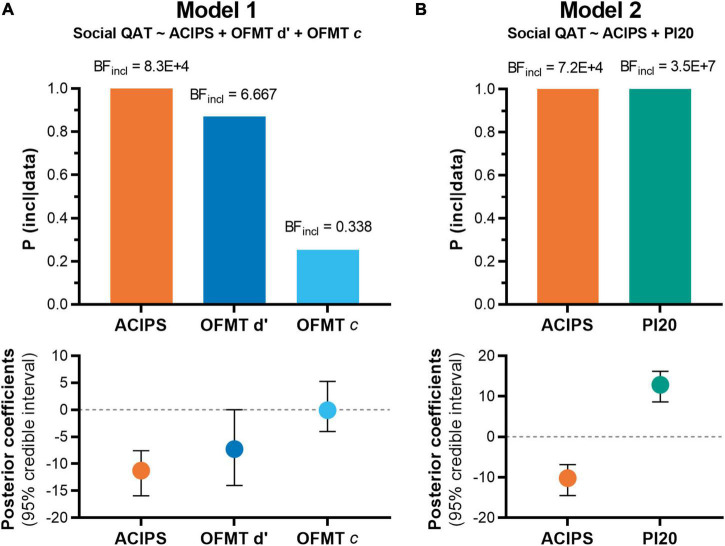
Inclusion probabilities and posterior coefficients for Bayesian linear regressions. **(A)** Shows the first analysis, containing the ACIPS together with face matching sensitivity (d′) and bias (c). The upper panel shows inclusion probabilities. The lower panel shows posterior coefficients, illustrating the large effect sizes of social anhedonia (low ACIPS score) and a smaller contribution by d′. **(B)** Shows the second analysis, which included self-reported prosopagnosia (PI20) instead of objective face matching measures. ACIPS, Anticipatory and Consummatory Interpersonal Pleasure Scale; BF, Bayes Factor; OFMT, Oxford Face Matching Task; PI20, 20-item Prosopagnosia Index.

Inclusion probabilities, inclusion BF (BF_*incl*_), and posterior β coefficients with credible intervals (Figure 4) confirmed high inclusion probabilities for face matching, social anhedonia, and prosopagnosia, but a relatively small effect size for OFMT d′ (Figure 4A, lower panel).

These results strongly support independent contributions of social anhedonia and poor face processing to autistic-like social function.

## 4 Discussion

We found that social anhedonia and prosopagnosia together explained about 44 % of the variance in social QAT, whereas mentalizing was less likely to contribute. Objectively measured face matching performance was a weaker predictor, but together with social anhedonia it explained almost a third of the variance in social QAT. These findings suggest that there is a close relationship of these constructs with social QATs, and that they are suitable as dimensional measures of autism-relevant social function.

The contribution of social anhedonia traits is consistent with previous research and with the social motivation theory of autism (Chevallier et al., 2012a; Berthoz et al., 2013; Novacek et al., 2016; Gadow and Garman, 2020), even though we cannot exclude influences by environmental factors, such as negative social experiences or adverse social-life outcomes (Mazurek, 2014; Jaya et al., 2022). Social motivation deficits in autism are indicated by reduced orienting to social stimuli and decreased reward-driven prosocial behaviors (Chevallier et al., 2012b). A meta-analysis of neuroimaging studies has provided support for atypical activation of reward circuitry to both social and non-social stimuli in autism (Clements et al., 2018), but there may also be an influence of higher-level control systems on the expression of anhedonia, possibly involving reduced coactivation of lateral prefrontal cortex to positive social input (Tully et al., 2014; Yin et al., 2015). The relative contributions of social and non-social brain networks, or of intrinsic reward valence versus associative learning, to social anhedonic traits are not known (Chevallier et al., 2012b). Thus, the observed correlation between social QATs and social anhedonia may reflect variations in both reward systems and higher-level systems. Social anhedonia is a transdiagnostic construct that is broadly associated with poor psychological outcomes and increased risk of psychosis (Chevallier et al., 2012a; Barkus and Badcock, 2019). However, it is not known whether social anhedonia predicts equally poor outcomes in people with high levels of QATs as it does in other populations (Barkus and Badcock, 2019). Regardless, mechanisms of social anhedonia could plausibly overlap with the mechanisms of autistic social function.

Despite the prominent status of mentalizing as a primary symptom of autism (Holt et al., 2014; Baron-Cohen et al., 2015), we found weak evidence for its contribution to social QATs. Previous studies have shown variable results. For example, RMET accuracy was correlated with social QATs in parents of autistic children (Losh et al., 2009), but was not significantly associated with QATs in other studies (Miu et al., 2012; Tajmirriyahi et al., 2013). Some studies using clinical populations have found a stronger deficit (Baron-Cohen et al., 2015; Waddington et al., 2018). This might suggest a more categorical pattern, but it could also be due to a small effect size that becomes more obvious when sampling more from the upper levels of the distribution of autistic-like traits. Correlations may also be attenuated by the relatively low internal reliability of the RMET (Olderbak et al., 2015). Thus, even if there is some association between mentalizing and QATs, it does not appear to be particularly robust when measured in this way.

We found strong evidence of the contributions of prosopagnosia to social QAT, and a smaller effect of face matching performance, consistent with previous research (Wilson et al., 2010; Halliday et al., 2014; Davis et al., 2017; Minio-Paluello et al., 2020; Gehdu et al., 2022; Stantić et al., 2022b). Brain imaging studies have suggested that the face network fails to develop normally in children with autism, resulting in weaker specialization for face stimuli (Joseph et al., 2015). It has been suggested that face representations in autistic individuals are less robust to variations in light, angle and other properties, possibly mediated by atypical low-level perception (Gehdu et al., 2022). Parents of children with autism performed worse than controls in a face memory tasks (Wilson et al., 2010), and showed hyperactivation of the fusiform face area and amygdala during face processing tasks (Yucel et al., 2015). Activation of the fusiform face area by face stimuli, measured by the N170 event-related potential, was linearly predicted by QATs in non-clinical samples (Lazar et al., 2014; Desai et al., 2019), and QATs were associated with altered blood-oxygen level dependent responses in the extended face network (Nummenmaa et al., 2012). The stronger QAT association with the self-report measure than the objective test might reflect distinct roles of face perception and face memory, as only the PI20 measures both (Stantić et al., 2022a). We believe that OFMT, despite the smaller effect size, provides a valuable paradigm as it measures a more circumscribed neural process in a more objective way than the PI20.

Our results suggest a likely association of social anhedonia and prosopagnosia with QAT-related social difficulties. Both social anhedonia and prosopagnosia have previously been linked to social difficulties independently of autism (Avery et al., 2016; Barkus and Badcock, 2019; Barkus, 2021). Which other constructs might explain the remaining variance in social QAT? One area that was not independently assessed in this study is pragmatic language, the ability to use language in social situations (Rapin and Dunn, 2003). Pragmatic language ability in itself is a broad construct, and some components of it are included in the RDoC matrix, including lower-level constructs such as “production of facial communication.” Higher-level components, such as irony perception, have not yet been fully specified in the matrix (Elvevåg et al., 2016). Another potential predictor of social QATs is social anxiety (Dickter and Burk, 2021), which is distinct from social anhedonia (Barkus and Badcock, 2019). Both social and generalized anxiety can be measured with self-report and may be a good addition to studies like ours. It would also be useful to collect dimensional measurement of psychopathological traits and states, such as depressive symptoms, as they may contribute to social symptoms. In addition, future research could investigate environmental factors which may play a role in shaping social abilities, including characteristics, such as QAT, of close family (Dickson et al., 2018).

We used the recently developed OFMT to investigate face matching ability, and found similar results to previous studies (Stantić et al., 2022a). However, we observed a previously unreported response bias in the OFMT, despite efforts to make sure that the same and different face pairs were equally difficult. During task development, Stantić et al. (2022a) used three independent face recognition algorithms to derive an average “similarity rating” for each pair of faces. The easiest same-face trials (similar photos of one person) and the most difficult different-face trials (similar photos of different people), and vice versa, were matched based on the algorithms’ similarity scores. However, our results showed a much lower probability of perceiving face pairs as matching in same-face trials compared with different-face trials. This may be interpreted as being consistent with differences in face processing between face recognition AIs and the human brain. However, as the processing strategies of the face recognition AIs are generally based on machine learning and largely unknown, we cannot readily interpret what exactly differs. It is possible that humans have developed a decision-making bias toward different-people judgments, but the bias may also be related to stimulus properties such as the lighting or viewpoint. Based on our findings, we recommend using d′ instead of the total accuracy to circumvent this discrepancy.

Future research may investigate specific constructs and their relationships to QATs across different units of analysis (Cuthbert and Insel, 2013), from genes and molecules to physiology and behavior. While this study integrates some measurements across behavioral paradigms and self-report, convergence with biological evidence will be necessary to reach a more detailed interpretation of our results. An experimental study replicating our results in tandem with e.g., brain imaging or polygenic risk scores, would provide stronger evidence for an endophenotypic relationship between social constructs. Future research can also expand focus to include the influence of variations in social constructs on other psychiatric disorders.

Our results indicate that a battery of RDoC-consistent, neurally based, tests may provide better “resolution” than classical QAT scales, which were validated against the diagnostic category (Baron-Cohen et al., 2001b; English et al., 2021). Such higher precision is particularly valuable for neuroscience studies aiming to find neural correlates of behavioral differences.

## 5 Conclusion

In conclusion, our findings suggests that social anhedonia and face identity perception contribute to social autistic-like traits. We propose that social difficulties in autism can be understood and investigated as a combination of quantifiable dimensional endophenotypes, each with their own specific objective measurements. By utilizing dimensional measurements, researchers can better understand how certain traits converge and form a larger construct, such as a psychiatric diagnosis, without the need to rely on diagnostic dichotomies.

## Data availability statement

The datasets presented in this study can be found in online repositories. The names of the repository/repositories and accession number(s) can be found below: Open Science Framework at https://osf.io/an9jm/.

## Ethics statement

Ethical review and approval was not required for the study on human participants in accordance with the local legislation and institutional requirements. The patients/participants provided their written informed consent to participate in this study.

## Author contributions

JFP designed and performed the research, analyzed the data, and wrote the manuscript. JW designed the research and wrote the manuscript. KI designed the research, analyzed the data, and wrote the manuscript. All authors contributed to the article and approved the submitted version.

## Footnotes

1
www.prolific.co

2
www.gorilla.sc

3
https://osf.io/weazf
