# Supplementary material for: Contributions of face processing, social anhedonia and mentalizing to the expression of social autistic-like traits
Source: Front Behav Neurosci. 2022 Dec 22;16:1046097. doi: 10.3389/fnbeh.2022.1046097 (PMC9817135; doi:10.3389/fnbeh.2022.1046097)
Supplement: Supplementary file 1 [file Table_1.DOCX]

Supplementary Material

**Table S1.**

Associations between social anhedonia and social quantitative autistic traits.

|  |  |  | **95% CI** | |  |  |  |
| --- | --- | --- | --- | --- | --- | --- | --- |
|  | **β** | **S.E.** | **Lower** | **Upper** | **Stand. β** | **t** | **p** |
| (Intercept) | 108.756 | 9.517 | 89.940 | 127.572 |  | 11.428 | 9.7×10^–22^ |
| Gender (male=1) | 0.538 | 2.697 | –4.795 | 5.871 |  | 0.199 | 0.842 |
| Ethnicity (white=1) | 1.453 | 4.520 | –7.483 | 10.389 |  | 0.322 | 0.748 |
| Mood disorder | 3.643 | 3.154 | –2.593 | 9.880 |  | 1.155 | 0.250 |
| Anxiety disorder | 1.680 | 3.218 | –4.682 | 8.041 |  | 0.522 | 0.602 |
| ADHD | –4.739 | 5.549 | –15.711 | 6.233 |  | –0.854 | 0.395 |
| **Age** | **–0.393** | **0.119** | **–0.629** | **–0.157** | **–0.269** | **–3.295** | **0.001** |
| **ACIPS** | **–0.511** | **0.093** | **–0.694** | **–0.328** | **–0.418** | **–5.515** | **1.6×10^–7^** |
| *Note.* Model: R^2^ = 0.248, F_7,139_ = 6.565, p = 1.1×10^–6^. Abbreviations: ACIPS, Anticipatory and Consummatory Interpersonal Pleasure Scale; ADHD, attention deficit/hyperactivity disorder. | | | | | | | |

**Table S2.**

Associations between prosopagnosia and social quantitative autistic traits.

|  |  |  | **95% CI** | |  |  |  |
| --- | --- | --- | --- | --- | --- | --- | --- |
|  | **β** | **S.E.** | **Lower** | **Upper** | **Stand. β** | **t** | **p** |
| (Intercept) | 21.621 | 8.379 | 5.055 | 36.188 |  | 2.581 | 0.011 |
| Gender (male=1) | 4.218 | 2.577 | –0.877 | 9.312 |  | 1.637 | 0.104 |
| Ethnicity (white=1) | 5.936 | 4.322 | –2.608 | 14.481 |  | 1.374 | 0.172 |
| Mood disorder | 5.425 | 2.998 | –0.503 | 11.354 |  | 1.809 | 0.073 |
| Anxiety disorder | 3.237 | 3.062 | –2.718 | 9.291 |  | 1.057 | 0.292 |
| ADHD | –5.724 | 5.280 | –16.164 | 4.715 |  | –1.084 | 0.280 |
| **Age** | **–0.332** | **0.112** | **–0.554** | **–0.110** | **–0.228** | **–2.962** | **0.004** |
| **PI20** | **0.883** | **0.128** | **0.630** | **1.135** | **–0.429** | **6.918** | **1.5×10^–10^** |
| *Note.* Model: R^2^ = 0.319, F_7,139_ = 9.284, p = 2.1×10^–9^. Abbreviations: PI20, 20-item Prosopagnosia Index; ADHD, attention deficit/hyperactivity disorder. | | | | | | | |

**Table S3.**

Associations between face matching performance and social quantitative autistic traits.

|  |  |  | **95% CI** | |  |  |  |
| --- | --- | --- | --- | --- | --- | --- | --- |
|  | **β** | **S.E.** | **Lower** | **Upper** | **Stand. β** | **t** | **P** |
| (Intercept) | 83.422 | 9.139 | 65.353 | 101.490 |  | 9.128 | 7.3×10^–16^ |
| Gender (male=1) | 0.760 | 2.940 | –5.053 | 6.574 |  | 0.259 | 0.796 |
| Ethnicity (white=1) | 3.596 | 4.885 | –6.063 | 13.255 |  | 0.736 | 0.463 |
| Mood disorder | 4.564 | 3.401 | –2.159 | 11.288 |  | 1.342 | 0.182 |
| Anxiety disorder | 3.311 | 3.485 | –3.579 | 10.202 |  | 0.950 | 0.344 |
| ADHD | –5.550 | 5.991 | –17.396 | 6.296 |  | –0.926 | 0.356 |
| **Age** | **–0.366** | **0.132** | **–0.627** | **–0.106** | **–0.251** | **–2.786** | **0.006** |
| **OFMT d′** | **–9.919** | **3.974** | **–17.775** | **–2.062** | **–0.214** | **–2.496** | **0.014** |
| *Note.* Model: R^2^ = 0.123, F_7,139_ = 2.793, p = 0.009. Abbreviations: OFMT, Oxford Face Matching Task; ADHD, attention deficit/hyperactivity disorder. | | | | | | | |
